# Inhibition Peroxiredoxin‐2 by Capsaicin Ameliorates Rheumatoid Arthritis via ROS‐Mediated Apoptosis in Fibroblast‐Like Synoviocytes

**DOI:** 10.1002/mco2.70209

**Published:** 2025-05-29

**Authors:** Hengkai He, Mingjing Hao, Piao Luo, Junhui Chen, Yehai An, Jingnan Huang, Ruiyi He, Qingfeng Du, Qian Zhang, Jigang Wang

**Affiliations:** ^1^ Guangdong Provincial Key Laboratory of Chinese Medicine Pharmaceutics School of Traditional Chinese Medicine and School of pharmaceutical Sciences Southern Medical University Guangzhou China; ^2^ State Key Laboratory of Southwestern Chinese Medicine Resource School of Pharmacy Chengdu University of Traditional Chinese Medicine Chengdu China; ^3^ Department of Pulmonary and Critical Care Medicine Shenzhen Institute of Respiratory Diseases and Shenzhen Clinical Research Centre for Geriatrics Shenzhen People's Hospital First Affiliated Hospital of Southern University of Science and Technology Second Clinical Medical College of Jinan University Shenzhen Guangdong China; ^4^ Guangdong Basic Research Center of Excellence for Integrated Traditional and Western Medicine for Qingzhi Diseases Guangzhou China; ^5^ State Key Laboratory for Quality Ensurance and Sustainable Use of Dao‐di Herbs Artemisinin Research Center and Institute of Chinese Materia Medica China Academy of Chinese Medical Sciences Beijing China

**Keywords:** activity‐based protein profiling, antioxidant, capsaicin, peroxiredoxin‐2, rheumatoid arthritis

## Abstract

Rheumatoid arthritis (RA), a prevalent and incurable autoimmune disease globally, is characterized by the immune system attacking the body's own tissues, leading to joint inflammation and damage. Capsaicin (CAP), from *Capsicum annuum L*., is known for its burning sensation‐inducing property and has shown various pharmacological effects, yet its specific mechanisms and targets in RA treatment remain largely unclear. This study aimed to investigate the role of CAP in RA by synthesizing CAP probes and using activity‐based protein profiling. We found that CAP reduced joint swelling in arthritic mice and exerted anti‐inflammatory and antiproliferative effects on fibroblast‐like synoviocytes. We identified that CAP binds to PRDX2, inhibiting its antioxidant function and inducing oxidative stress and apoptosis, contributing to the antiarthritic effects. These results suggest that PRDX2 is a potential target for CAP in RA treatment, providing new insights into the molecular mechanisms and potential therapeutic strategies for RA.

## Graphical abstract

Capsaicin attenuating arthritis by inhibiting AKT phosphorylation and activating the NRF2–HO‐1 axis via targeting PRDX2

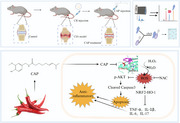

## 1 Introduction

Rheumatoid arthritis (RA) is a common autoimmune disorder distinguished by inflammatory and edematous joints, affecting approximately 1% of the global population with a higher incidence in older adults [1, 2, 3]. The pathogenesis of RA involves the immune system aberrantly attacking its own cells, leading to inflammation and the thickening of the synovium within joints, ultimately results in irreversible damage to bone and cartilage [1]. Key cellular players in RA include macrophage‐like synoviocytes and fibroblast‐like synoviocytes (FLS), where are integral components of the articular canal's lumen layer [4]. These cells are primarily responsible for secreting hyaluronic acid and phagocytosing exfoliated chondrocytes [4]. In RA, resident and infiltrating immune cells secrete inflammatory cytokines like TNF‐α, IL‐1β, and IL‐6 which activate pathological FLS [5]. This activation leads to synovial hyperplasia and the secretion of matrix metalloproteinases (MMPs) and additional inflammatory mediators, culminating in the destruction of synovial tissue and bone [6]. Therefore, targeting the abnormal activation of FLS may be crucial for effective RA treatment [7].

Natural products’ diverse bioactivities make target identification a strategic priority in drug discovery [8]. Capsaicin (CAP), the pungent phytochemical from chili peppers, serves dual roles as sensory stimulant and therapeutic agent. Beyond TRPV1‐mediated analgesia, its anti‐inflammatory, anticancer, and metabolic benefits suggest undercharacterized molecular targets, warranting systematic target deconvolution [9, 10, 11, 12]. Moderate amounts of reactive oxygen species (ROS) may promote tumor growth [13]. Interestingly, FLS seems to benefit from this high ROS environment [5]. Some drugs for the treatment of tumors such as paclitaxel and trisenox can lead to excessive elevation of ROS, such that the antioxidant system is unable to remove peroxides, resulting in apoptosis. This provides us with a new idea for the management of RA [14, 15, 16].

Peroxiredoxin‐2 (PRDX2) is an antioxidant enzyme belonging to the peroxiredoxin (PRDXs) family [17]. This protein family catalyzes the reduction of peroxides and scavenges ROS. PRDX2 demonstrates ubiquitous tissue distribution and critically regulates cellular homeostasis through modulation of proliferation, apoptosis, and differentiation. Chemotherapy‐induced hydrogen peroxide accumulation triggers cancer cell damage, a process effectively counteracted by PRDX2 overexpression. This mechanistic evidence establishes PRDX2 suppression as a potential therapeutic strategy. Clinically, upregulated PRDX2 mRNA levels in RA synovial fibroblasts show significant correlation with inflammatory disease pathogenesis through redox imbalance [18, 19]. This indicates that PRDX2 may play a crucial role in the inflammatory processes associated with RA.

Activity‐based protein profiling (ABPP) utilizes active site‐directed chemical probes to map functional enzymes in complex proteomes, enabling mechanistic dissection of enzymatic regulation and drug–target interactions. Our prior ABPP studies have successfully uncovered molecular targets of CAP, celastrol, and glaucocalyxin A in diverse disease models [20, 21, 22].

In our study, we explored the effects of CAP on arthritis progression using the collagen‐induced arthritis (CIA) model. We synthesized CAP probes and employed ABPP to identify PRDX2 as a potential therapeutic target. Our findings indicate that CAP binds to the cysteine residues of PRDX2, inhibiting its activity and expression, thereby preventing the removal of ROS and inducing oxidative stress and apoptosis. These results suggest that inhibiting PRDX2 with CAP could offer a novel therapeutic avenue for RA treatment.

## 2 Results

### 2.1 CAP Administration Reduces Arthritis Severity in CIA mice

**FIGURE 1:**
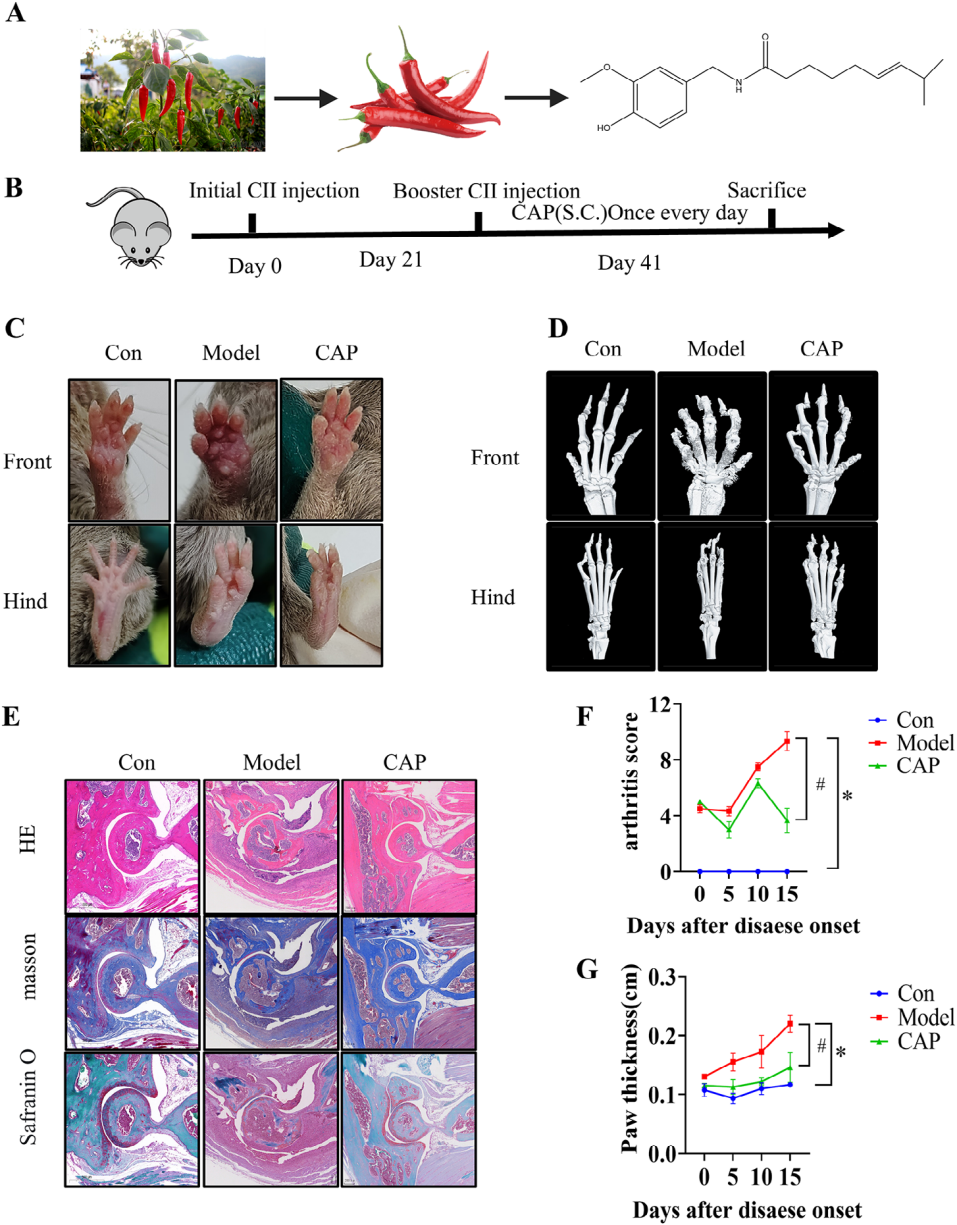
CAP treatment reduces the severity of arthritis in the CIA model. (A) The plant *Capsicum annuum L*. and the molecular structure of CAP. (B) Experimental timeline depicting arthritis induction and treatment protocol of CAP. (C, D, and E) Photographs and X‐rays of the ankle joint are provided, as well as section staining. (scale bar = 500 µm). (F and G) The increased ankle joint volume and the arthritis scores are shown in F and G. Data are shown as mean ± SD. Two‐way ANOVAD was used to analyze differences between groups, *p < 0.05, *n* = 3.

Animal experiments demonstrated CAP's antiarthritic efficacy in CIA mice. Arthritis induction was confirmed at day 21 postimmunization (Figure 1B). Model mice exhibited severe paw erythema/stiffness (vs. controls), which CAP treatment alleviated (Figure 1C). CAP significantly lowered arthritis scores and joint swelling (Figure 1F,G). Bone erosion and destruction are hallmark features of RA [23]. CT imaging showed CAP treatment significantly reduced bone destruction versus model group (Figure 1D). Synovial tissue analysis via hematoxylin‐eosin (H&E) staining revealed attenuated synovial hyperplasia in CAP‐treated mice. Joint histology (Masson/safranin O‐fast green staining) demonstrated CAP mitigated bone erosion and cartilage degradation (Figure 1E). Concurrently, CAP decreased leukocyte differential counts in blood (Figure S1). These findings collectively confirm CAP's efficacy in alleviating arthritis severity in CIA models.

### 2.2 CAP Inhibits the Production of Inflammatory Cytokines in the FLS and Macrophage Coculture System

**FIGURE 2:**
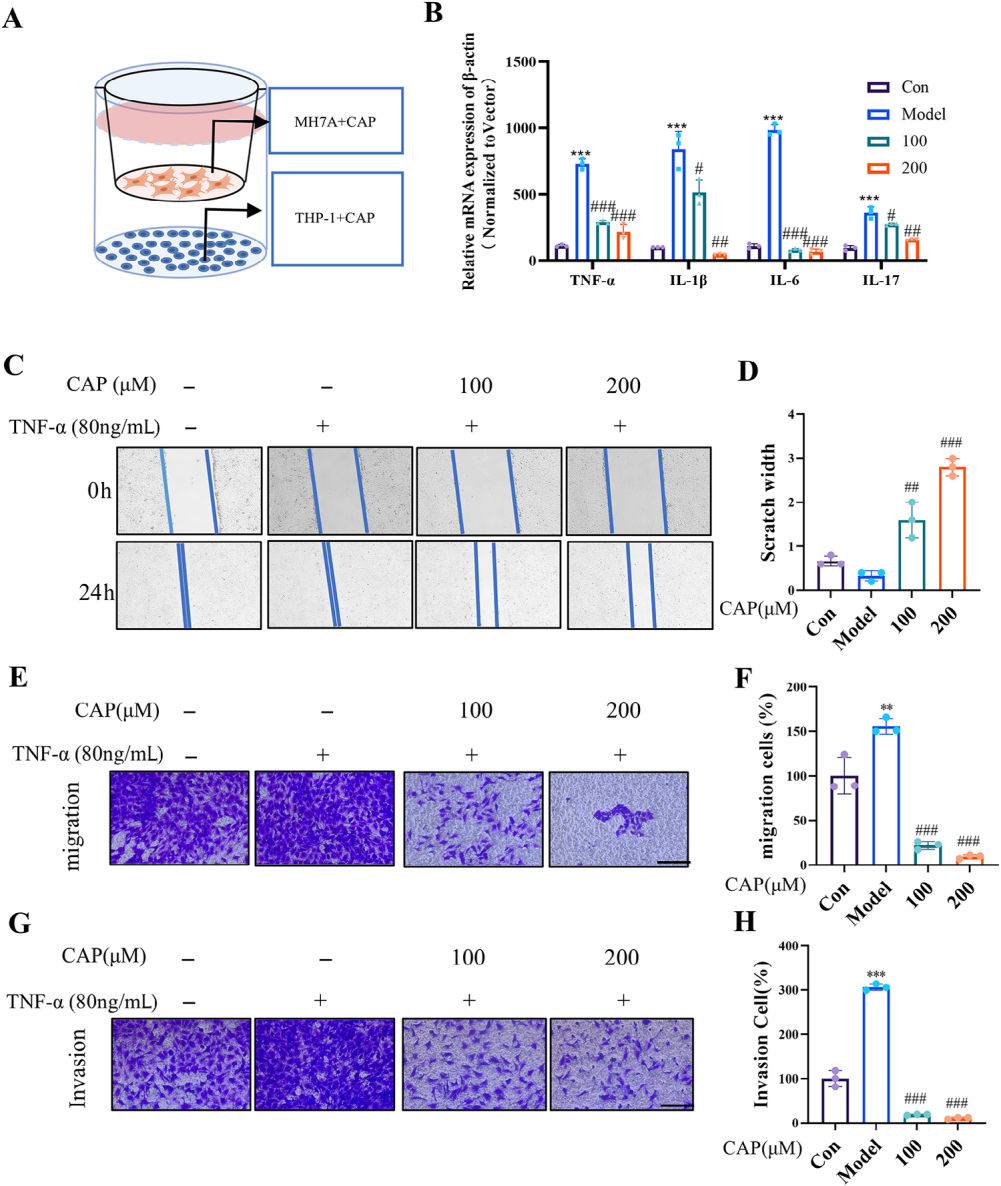
CAP inhibits inflammation of FLS cells. CAP inhibits inflammation of FLS cells. (A) Experimental design diagram of coculture THP‐1 with MH7A free of direct contact. (B) The total mRNA level of TNF‐α, IL‐1β, IL‐6, and IL‐17 in coculture of THP‐1 and MH7A cells. (C and D) Results and statistical analysis of the effect of CAP on TNF‐α‐induced MH7A cells in scratch assay. (E–H) Antimigration (E and F) and anti‐invasion (G and H) effects of CAP on MH7A cells (scale bar = 100 µm). The positive cell number was calculated using the Image J software. (*p < 0.05, **p < 0.01, **p < 0.001 vs. control; #*p* < 0.05, ##*p* < 0.01, ###*p* < 0.001 vs. si‐NC or model; *n* = 3).

FLS play a crucial role as pathogenic cells in RA [24]. Currently, there are limited reports on the use of CAP in RA treatment, although its anti‐inflammatory properties on RAW 264.7 cells were demonstrated in our previous study [25]. We established a THP‐1/MH7A (human FLS cell line) coculture system (Figure 2A) to simulate the RA inflammatory microenvironment. Under LPS/IFN‐γ stimulation, THP‐1 cells markedly upregulated TNF‐α, IL‐1β, IL‐6, and IL‐17 mRNA expression in MH7A cells, which was significantly suppressed by CAP treatment (Figure 2B). A RAW 264.7 macrophage/rat FLS coculture model further validated CAP's anti‐inflammatory effects (Figure S2F). Consistent results from animal studies and coculture experiments suggest CAP's anti‐inflammatory phenotype exhibits universality across cell types.

To explore whether CAP also possesses antiarthritic effects in vitro, we first selected 80 ng/mL TNF‐α‐induced MH7A cells for our experiments to trigger inflammation in vitro. We further estimated the anti‐inflammation effect of CAP after TNF‐α stimulation, and the results showed that CAP could inhibit NO level in cells (Figure S2A,B).

CAP demonstrated promising inhibitory effects on synovial invasion in animal models, which prompted us to conduct follow‐up experiments to assess cell scratch assays, migration, and invasion. These subsequent experiments aim to elucidate the mechanisms underlying the differential responses of FLS to CAP treatment.

### 2.3 CAP Significantly Inhibits the Aberrant Proliferation of MH7A

The development of RA typically involves the abnormal proliferation of FLS [26, 27]. In RA, this proliferation leads to inflammatory infiltration of the joints [28]. Moreover, the activation of FLS during inflammation results in the secretion of various inflammatory cytokines, which contribute to bone destruction and damage [29]. This underscores the importance of targeting the abnormal proliferation of FLS to manage RA.

In the cell scratch assay, while control and TNF‐α‐treated groups showed near‐complete wound closure, CAP treatment concentration‐dependently inhibited healing (Figure 2C,D). Transwell assays further demonstrated CAP's significant suppression of MH7A cell migration and invasion (Figure 2E–H). These findings indicate CAP's therapeutic potential in RA by targeting pathological FLS activities.

### 2.4 ABPP Reveals CAP Directly Targets to PRDX2

**FIGURE 3:**
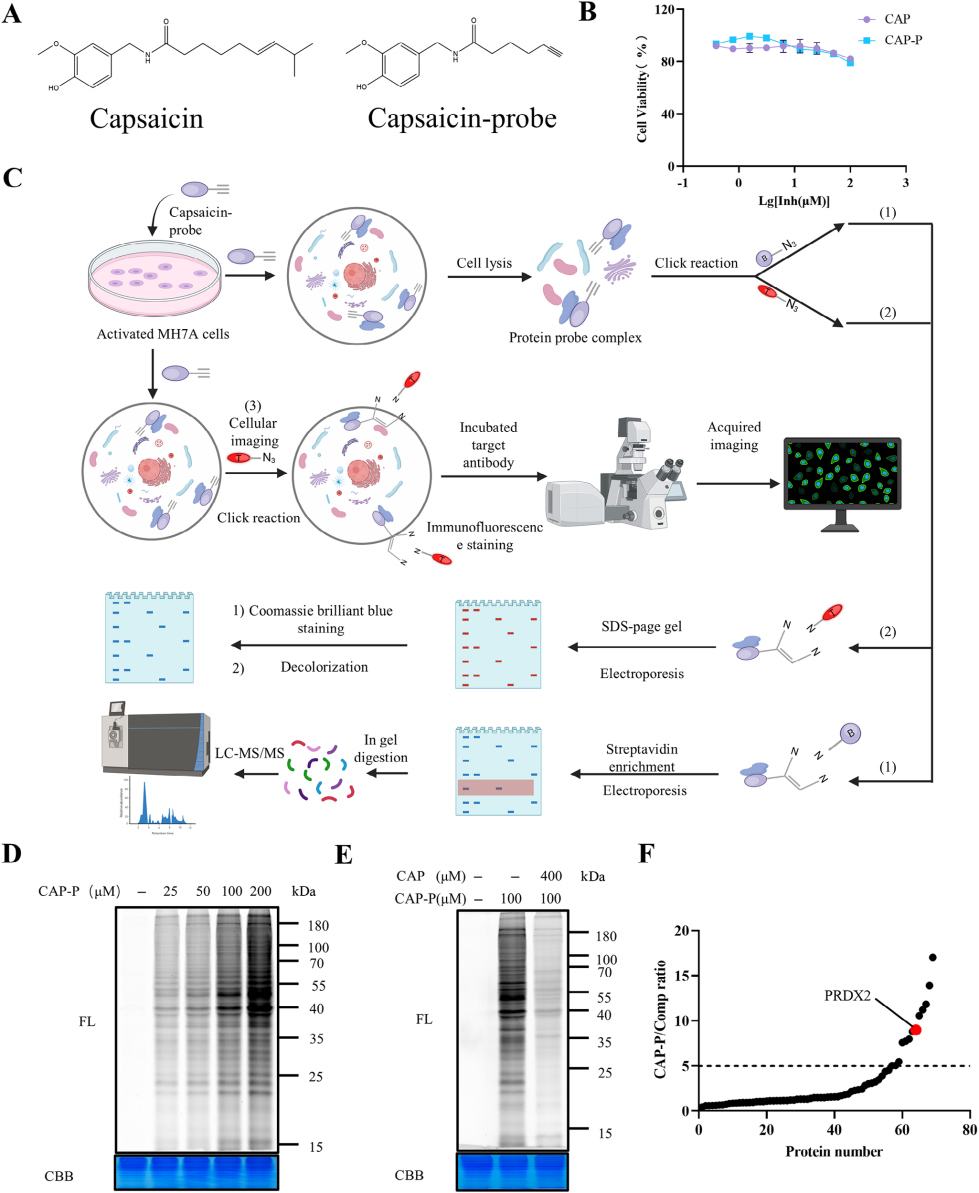
The ABPP method, combined with LC–MS/MS, was used to profile and identify the targets of CAP. (A) Molecular configuration of the capsaicin probe. (B) Cytotoxicity assay of CAP on MH7A cells (*n* = 3). (C) Comprehensive workflow for ABPP‐based profiling of potential CAP targets. (D) Dose‐dependent in situ protein labeling with CAP‐P in MH7A cells; (E) CAP competes with CAP‐P for in situ protein labeling; (F) bar graph illustrating the varying levels of enrichment for selected target proteins in the CAP‐P versus “compete” group, with each point indicating a calculated ratio value of CAP‐P to compete (mean ± SD, *n* = 3).

**FIGURE 4:**
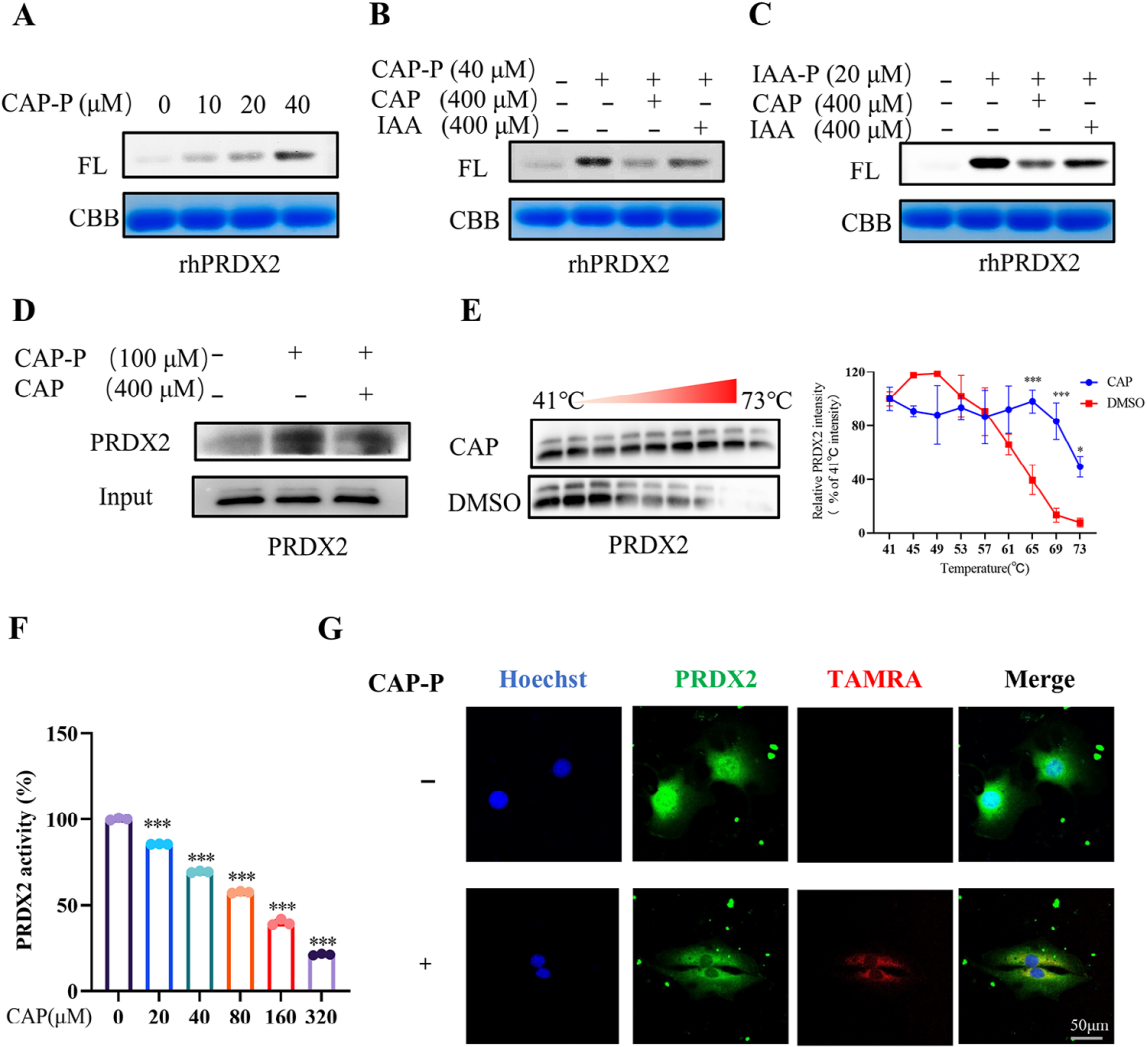
CAP binds to PRDX2 and inhibits its oxidative activity and expression level. (A–C) (A) Labeling of rhPRDX2 with CAP‐P. The competition for rhPRDX2 labeling involved (B) CAP‐P or (C) IAA‐yne, either with or without unmodified CAP or the cysteine‐alkylating agent IAA. (D) A CAP‐P pull‐down assay was conducted, followed by a western blot to verify the targeting of capsaicin to PRDX2 proteins in situ. (E) The interactions between capsaicin and PRDX2 were validated through CETSA‐WB. (F) CAP concentration‐dependent inhibits the activity of rhPRDX2; (F) peroxide‐degrading enzyme. (G) Colocalization of PRDX2 (green) with CAP‐P (red) on MH7A cells (scale bar = 50 µm) (*p < 0.05, **p < 0.01, **p < 0.001 vs. control; #*p* < 0.05, ##*p* < 0.01, ###*p* < 0.001 vs. model; *n* = 3).

To identify the target protein of CAP in its action against arthritis, we developed a chemical probe based on CAP, referred to as CAP probe (CAP‐P), as illustrated in Figure 3A [25]. CAP‐P exhibited comparable bioactivity to CAP (Figure 3B). Probe labeling optimization in MH7A cells revealed concentration‐dependent efficiency, establishing 100 µM CAP‐P as optimal (Figure 3D). Competitive assays confirmed target specificity through CAP dose‐responsive inhibition (Figure 3E). This validated protocol was applied for subsequent tandem mass tag (TMT) pull down studies (Figure 3C). Following the pull‐down, the probe‐labeled targets were quantified using TMT labeling. The proteins were then digested on‐bead, and the resulting peptide fragments were analyzed by mass spectrometry. This analysis successfully pinpointed PRDX2 as a direct interaction target of CAP (Figure 3F). This study elucidates CAP's antiarthritic mechanism via PRDX2 targeting, revealing therapeutic potential for arthritis. Validation experiments with recombinant human PRDX2 (rhPRDX2) demonstrated dose‐dependent CAP‐P labeling (Figure 4A), confirming direct CAP–PRDX2 interaction. To investigate CAP–PRDX2 binding specificity, rhPRDX2 was pretreated with excess CAP or cysteine‐targeting iodoacetamide (IAA), followed by CAP‐P labeling and TAMRA‐azide click visualization (Figure 4B). Competitive binding assays using alkyne‐functionalized IAA‐yne revealed CAP competes for cysteine residues on rhPRDX2 (Figure 4C), confirming CAP's cysteine‐dependent interaction with PRDX2. These results establish PRDX2's redox‐active cysteines as critical mediators of CAP's antiarthritic effects, advancing PRDX2‐targeted therapeutic strategies for RA.

Our studies revealed CAP selectively inhibits PRDX2 enzymatic activity in a dose‐dependent manner (Figures 4F and S3E). Pull‐down/Western blot assays demonstrated PRDX2‐specific binding, competitively displaced by excess CAP, implicating cysteine involvement (Figure 4D). CETSA‐WB analysis showed CAP binding enhances PRDX2 thermal stability (Figure 4E), suggesting structural stabilization. Intracellular colocalization of CAP‐P and PRDX2 (Figure 4G) confirmed direct interaction, while PRDX1 exhibited no activity inhibition (Figure S3E) despite in vitro labeling potential, with pull‐down assays excluding intracellular interaction (Figure S3A–D). These data establish CAP covalently targets PRDX2 via cysteines, suppressing its redox activity to mediate antiarthritic effects, positioning PRDX2 as a precision therapeutic target with minimized off‐target risks.

### 2.5 Prediction of CAP and PRDX2 Binding Sites

**FIGURE 5:**
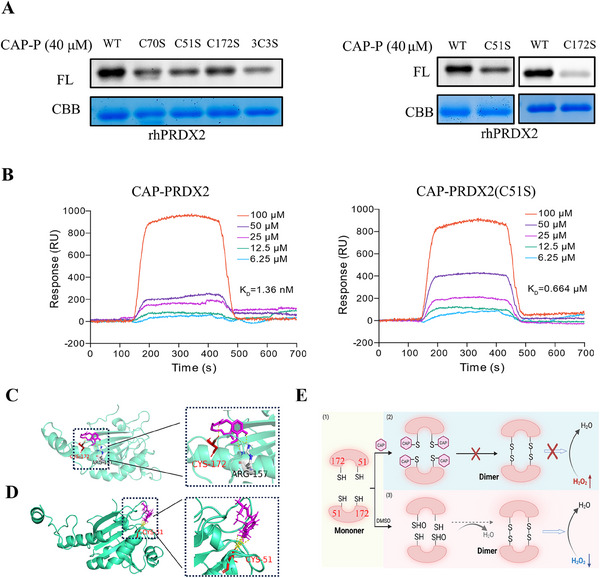
Prediction of CAP and PRDX2 binding sites. (A) Labeling of CAP‐P and PRDX2 wild‐type and mutant strains. (B) SPR experiments showed that the binding ability of Prdx2 and CAP decreased after the mutation of Cys51. (C and D) Molecular docking of CAP and PRDX2. (E) CAP inhibits PRDX2 dimer formation by binding to Cys172/51.

To investigate the precise interaction sites between CAP and PRDX2, we engineered mutant versions of PRDX2 with mutations at the sites Cys51, Cys70, and Cys172. The labeling experiments indicated that the binding capacity of CAP‐P to these mutants was markedly diminished, implying that these cysteines might be the critical binding sites for CAP and PRDX2 (Figure 5A). In addition, SPR experiments showed that mutations in Cys51/70 significantly reduced the stability of PRDX2 binding to CAP (Figures 5B and S4B). Molecular docking studies on PRDX2 revealed that the binding sites for CAP include the Cys172 and Cys51 residues (Figure 5C,D), corroborating our earlier findings. Notably, Cys172, a catalytic cysteine in PRDX2, is essential for completing antioxidant processes. CAP could be forming a covalent bond at the Cys172 site on PRDX2, thus blocking its ability to break down peroxides. Cys51 in Prdx2 interacts with H_2_O_2_ and subsequently forms a disulfide bond with Cys172 located on the second subunit of the dimer. Consequently, the binding of PRDX2 with CAP at the Cys51 and Cys172 sites could potentially disrupt its catalytic cycle, leading to an accumulation of H_2_O_2_ as illustrated in Figure 5E [30]. In addition, CAP may generate different oxidation products under oxidative conditions, which are involved in the binding of PRDX2 (Figure S4C).

### 2.6 Proteomic Analysis Showed that CAP Exerted Antiarthritic Effects by Inducing Oxidative Stress and Apoptosis

**FIGURE 6:**
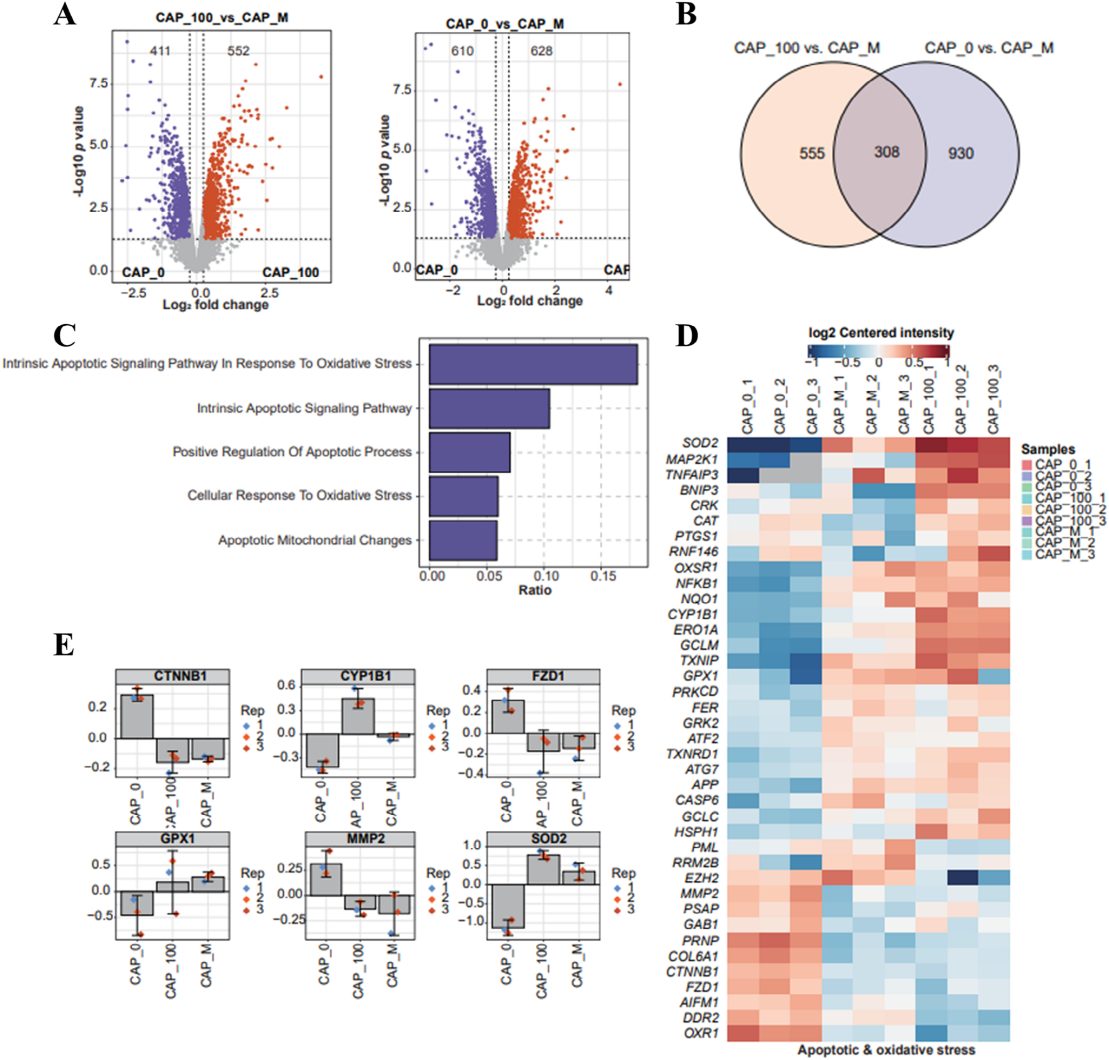
Proteomic analysis showed that CAP exerted antiarthritic effects by inducing oxidative stress and apoptosis. (A) Volcano gram analysis was performed on differential genes. (B) The overlap of differentially expressed proteins in CAP_100 versus CAP_M and CAP_0 versus CAP_M comparisons is demonstrated. (C) The enrichment of differentially expressed proteins in different biological pathways was demonstrated. (D) The expression of differentially expressed proteins in different treatment groups (CAP_0, CAP_100, and CAP_M) was demonstrated. (E) The expression changes of several key proteins (e.g., CTNNB1, CYP1B1, FZD1, GPX1, MMP2, and SOD2) in different treatment groups were demonstrated.

To investigate the anti‐inflammatory and antiproliferative effects of CAP on MH7A cells, we incubated the cells with 100 µM CAP. After incubation, the cells were collected for proteomic analysis. The results, as depicted in the provided figures, indicated significant activation of proteins associated with oxidative stress and apoptotic pathways following CAP treatment compared with the model group. Specifically, the volcano plots and Venn diagram demonstrate differential protein expression, while the bar chart highlights the enrichment of pathways related to intrinsic apoptotic signaling in response to oxidative stress (Figure 6A–C). The heatmap further illustrates changes in protein expression levels, with notable upregulation of proteins such as SOD2 and MAP2K1 (Figure 6D). Additionally, individual protein expression profiles, such as CTNNB1, CYP1B1, and FZD1, confirm these trends (Figure 6E). Subsequently, we will conduct a detailed mechanism analysis to elucidate these pathways.

### 2.7 CAP Induces an Increase in ROS Levels in MH7A Cells

**FIGURE 7:**
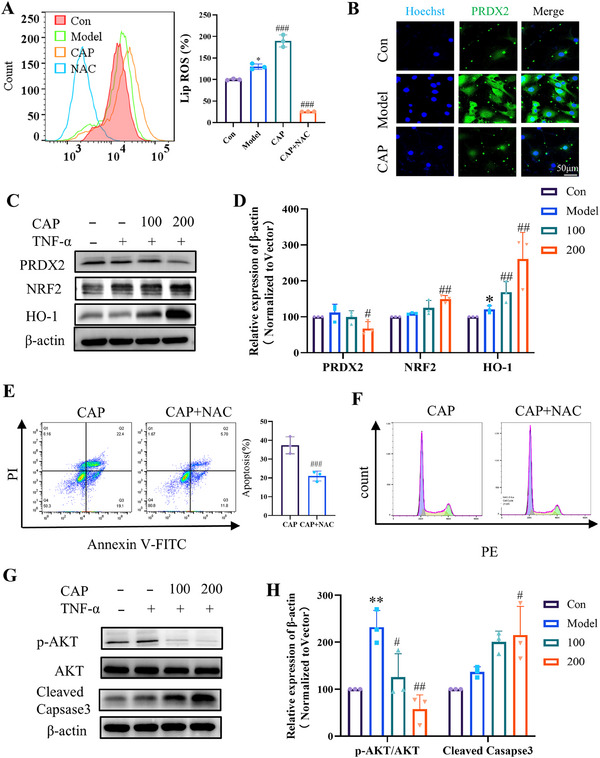
In vitro mechanistic study of CAP's inhibition of MH7A cells. (A) ROS assay by flow cytometry assay. (B) Immunofluorescence was used to detect the expression of PRDX2 after CAP treatment. (C and D) Western blotting analysis of PRDX2, HO‐1, and NRF2 expression in MH7A treated with TNF and CAP for 24 h. (E) Apoptotic assay by flow cytometry assay. (F) Cell cycle assay by flow cytometry assay. (G and H) Western blotting analysis of p‐AKT, AKT, and cleaved caspase‐3 expression in MH7A treated with TNF and CAP for 24 h. (*p < 0.05, **p < 0.01, **p < 0.001 vs. control; #*p* < 0.05, ##*p* < 0.01, ###*p* < 0.001vs. model; *n* = 3).

To investigate the potential mechanism behind CAP's antiproliferative effects, we conducted ROS testing and observed that CAP resulted in a marked rise in intracellular ROS levels, an effect that was reversed by N‐acetylcysteine (NAC), a cysteine prodrug known for its lipophilic antioxidant properties (Figure 7A). Subsequently, we employed fluorescence imaging and Western blot to detect changes in PRDX2 protein expression following CAP treatment. Our results showed a significant inhibition of PRDX2 expression after CAP treatment (Figure 7B–D).

The NRF2/HO‐1 pathway is generally regarded as a critical component of cellular protective mechanisms that counteract oxidative stress and inflammation. Following this, we investigated the NRF2/HO‐1 pathway, a classical mechanism involved in oxidative stress. Our findings revealed that CAP significantly upregulated the expression of both NRF2 and HO‐1 proteins. This suggests that the CAP‐induced increase in ROS levels activates the NRF2/HO‐1 pathway (Figure 7C,D).

### 2.8 CAP Induces Apoptosis in MH7A

To investigate the potential mechanism behind CAP antiproliferative effects, we used propidium iodide (PI) and FITC staining to analyze MH7A cells via flow cytometry. The results indicated that CAP significantly induced apoptosis in these cells, an effect that was reversed by NAC (Figure 7E). These results suggest that an excess of ROS induces apoptosis in MH7A cells. Additionally, cell cycle analysis revealed that CAP inhibited the progression of the cell cycle in MH7A cells at the G2 phase, with NAC reversing this cycle arrest (Figures 7F and S6C). These findings imply that CAP may inhibit the proliferation of MH7A cells by suppressing PRDX2 activity, consequently inducing an increase in intracellular ROS, which in turn leads to apoptosis and cell cycle arrest.

Furthermore, we examined changes in AKT protein and found that CAP inhibited the phosphorylation of AKT and increased the expression of cleaved caspase‐3 (Figure 7G,H). These findings suggest that CAP directly interacts with and suppresses the function of PRDX2 in MH7A cells. On one hand, this interaction results in a rise in ROS and activates the NRF2/HO‐1 axis, exerting anti‐inflammatory effects. On the other hand, it inhibits the phosphorylation of the AKT pathway, leading to an increased expression of cleaved caspase‐3, which ultimately causes cell cycle arrest and apoptosis, thereby exerting antiproliferative effects.

### 2.9 Inhibition of PRDX2 Enhances CAP‐Induced Inhibition of Proliferation and Apoptosis

To functionally verify whether PRDX2 is a key protein in CAP‐induced apoptosis, we designed and synthesized shRNA targeting PRDX2. We then used shRNA to knock down PRDX2 in MH7A cells and conducted apoptosis flow cytometry analysis (Figure S5A–D). Similar to the effect of CAP, PRDX2 knockdown led to increased apoptosis in MH7A cells. This demonstrates that reduced PRDX2 expression can promote apoptosis in MH7A cells.

**FIGURE 8:**
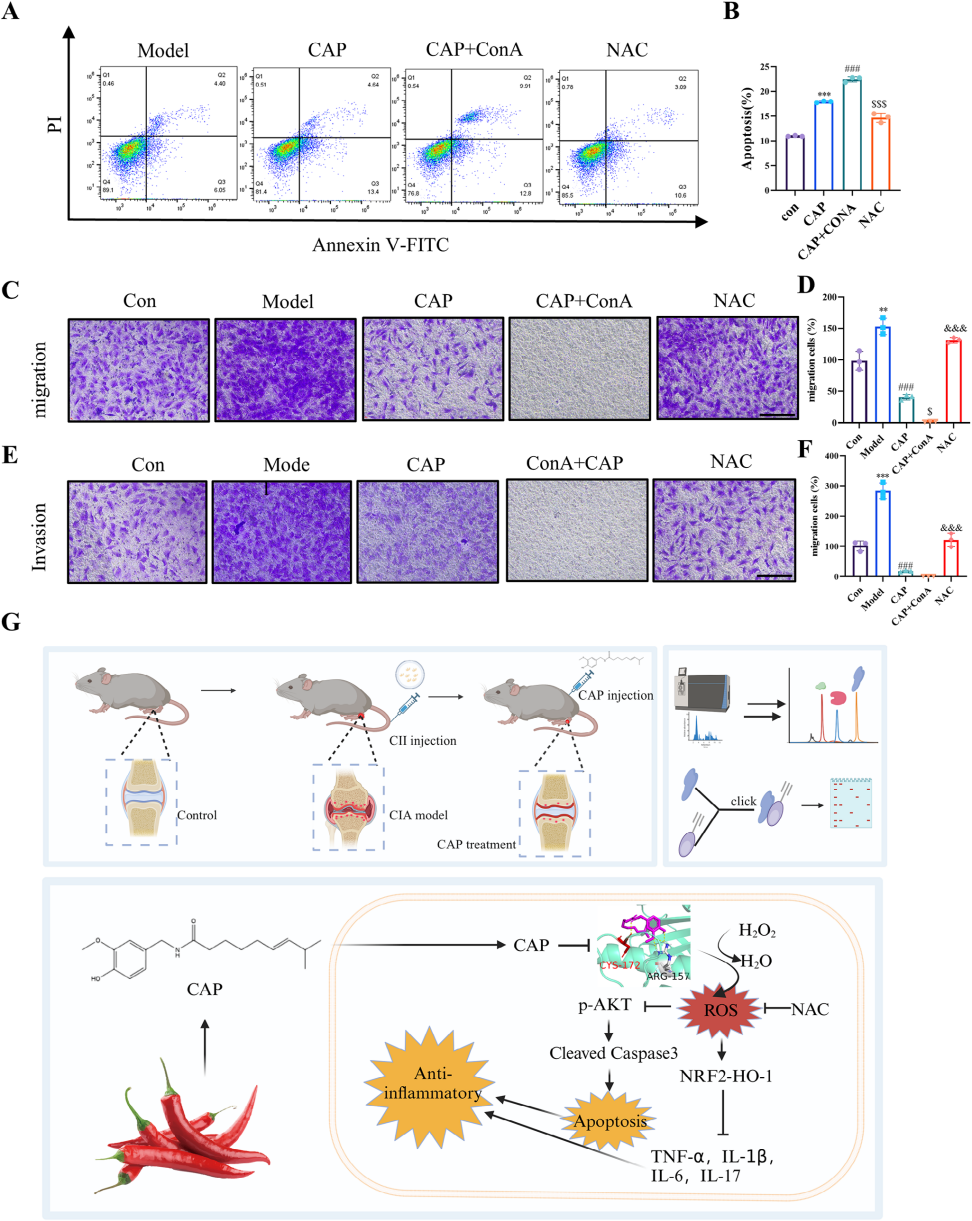
CAP ameliorated RA by promoting apoptosis. (A and B) Apoptotic assay by flow cytometry assay. (C–F) Antimigration (C and D) and anti‐invasion (E and F) of CAP on MH7A cells (scale bar = 100 µm). (G) Schematic diagram of capsaicin attenuating RA by inhibiting AKT phosphorylation and activating the NRF2–HO‐1 axis. (*p < 0.05, **p < 0.01, **p < 0.001 vs. control; #*p* < 0.05, ##*p* < 0.01, ###*p* < 0.001 vs. model; $*p* < 0.05, $$*p* < 0.01, $$$*p* < 0.001 vs. CAP, &*p* < 0.05, &&*p* < 0.01, &&&*p* < 0.001 vs. CAP + ConA, *n* = 3).

Given the insufficient silencing efficiency of PRDX2, we utilized the PRDX2 inhibitor Conoidin A (ConA) in combination with CAP. Apoptotic analysis using flow cytometry with PI and FITC staining revealed that various concentrations of ConA induced apoptosis in MH7A cells, with 2.5 µmol/L selected as the optimal concentration for subsequent experiments (Figure S5E,F). Building on this, we conducted an apoptosis assay combining ConA with CAP and observed that the apoptosis induced by this combination was statistically significant compared with the single treatment groups, and it could be reversed by NAC, indicating a synergistic effect between the two (Figure 8A,B).

Further, we replicated the scratch, migration, and invasion assays initially prompted by CAP using ConA and found that the combination of CAP and ConA significantly inhibited the abnormal proliferation and activation of MH7A cells, effects that were also reversible by NAC (Figure 8C–F). Collectively [31], these finding suggest inhibition of PRDX2 exacerbates the effects of CAP‐induced apoptosis and the inhibition of cell proliferation, highlighting the critical role of PRDX2 in these cellular processes.

## 3 Discussion

RA is a chronic and intractable autoimmune disorder that afflicts a significant number of individuals worldwide. The current treatment modalities, such as nonsteroidal anti‐inflammatory drugs and methotrexate, are often accompanied by adverse effects, particularly in the digestive and renal systems [4, 32]. Moreover, the high cost of biologics renders them inaccessible to many patients, and their effectiveness is not absolute [33]. Prolonged RA can lead to a decline in work productivity, job loss, and even disability [34, 35]. Consequently, the development of novel, efficacious, affordable, and less toxic therapies is of utmost urgency in the field of arthritis treatment. Synovial hyperplasia and dysfunction are central to the pathogenesis of RA, ultimately resulting in bone erosion, joint deformity, and fibrosis [36, 37].

CAP, the principal active component of *Capsicum annuum L*., has been traditionally employed for pain relief in arthritic conditions [37, 38, 39]. To unravel the antiarthritic target of CAP, we designed and synthesized a novel CAP probe conjugated with an alkyne tag [22, 40, 41]. In this study, we leveraging ABPP technology, in conjunction with LC–MS/MS and proteomic analysis, we identified PRDX2 as a direct binding target of CAP. Subsequent experimental validation, including pull‐down assays, CETSA‐WB, rhPRDX2 labeling, and molecular docking studies, confirmed the specific binding of CAP to the cysteine residues (Cys51 and Cys172) of PRDX2, leading to the inhibition of its enzymatic activity.

Accumulating evidence suggests that PRDX2 overexpression is implicated in tumorigenesis and the progression of various inflammatory diseases, including psoriasis, inflammatory bowel disease, and RA. Inhibition of PRDX2 may thus represent a promising therapeutic strategy for autoimmune inflammatory disorders [18, 42, 43, 44, 45].

The phosphatidylinositol 3‐kinase (PI3K)/AKT signaling pathway is a crucial intracellular signaling cascade that regulates cell proliferation, metabolism, angiogenesis, and survival in response to extracellular stimuli [46, 47, 48, 49]. Inhibition of AKT phosphorylation has therefore emerged as a potential therapeutic approach for RA [50, 51]. Our results showed that CAP treatment dose‐dependently inhibited AKT phosphorylation and induced apoptosis in FLS, highlighting its potential to counteract the abnormal proliferation of FLS.

The NRF2/HO‐1 pathway is a well‐characterized cellular defense mechanism against oxidative stress [52, 53]. In the presence of oxidative stress or other pathological insults, NRF2 dissociates from Keap1, translocates to the nucleus, and forms a transcriptional complex with Maf and Jun proteins to activate a battery of antioxidant and anti‐inflammatory genes, including HO‐1 [54, 55, 56, 57, 58, 59, 60].

CAP induced compensatory NRF2/HO‐1 upregulation via PRDX2 inhibition‐mediated oxidative stress, potentially underpinning its RA anti‐inflammatory effects. Study limitations include: (1) unvalidated target gene overexpression (possibly due to MH7A cell transfection inefficiencies), requiring alternative delivery approaches; (2) NRF2/HO‐1 pathway complexity impeding direct genetic modulation; (3) lack of transgenic in vivo validation models.

## 4 Conclusion

In this study, we utilized ABPP technology in conjunction with proteomics to explore the mechanisms by which CAP modulates inflammation and apoptosis. Our findings demonstrate that CAP directly binds to PRDX2, leading to its inhibition. This interaction results in an increase in ROS, which subsequently activates the NRF2/HO‐1 signaling pathway. Activation of this pathway influences the activity of the AKT protein, leading to the inhibition of inflammatory cytokines and induction of apoptosis. Those insights provide a novel perspective on the molecular interactions of CAP and present a promising avenue for the development of new therapeutic strategies for the treatment of RA. By targeting PRDX2, a key regulator of oxidative stress and inflammation, CAP offers a potential mechanism for modulating disease pathways in a manner that could minimize side effects associated with current RA treatments. This approach could lead to the development of safer, more effective drugs that provide relief for patients suffering from this debilitating condition.

## 5 Materials and Methods

### 5.1 Reagents

CAP (≥98%; Shanghai yuanye Bio‐Technology); penicillin–streptomycin (100×), Glutamax, trypsin (0.25%), DMEM/RPMI‐1640 (Gibco, US); FBS (Excell, China); NaVc, THPTA, CuSO4, TAMRA/biotin azides (Sigma, USA); PRDX2/β‐actin/HMGB1/caspase‐3 antibodies (Proteintech, China); p‐AKT/AKT antibodies (Abcam, UK); other chemicals (Sigma) were used.

### 5.2 Cell Culture

In this study, we employed the immortalized human FLS cell line MH7A as our in vitro experimental model. MH7A cells were kindly provided by Professor Lixia Yuan of Southern Medical University. Raw 264.7 cells were sourced from the Chinese National Cell Bank (Beijing, China). Rat FLS (RFLS) were isolated from rat knee joints. THP‐1 cells were purchased from Procell (Wuhan, China).

### 5.3 Animal Experiments

#### 5.3.1 Ethical Approval

This study received approval from the Care and Use of Laboratory Animals Center at Shenzhen People's Hospital, ensuring adherence to ethical standards for animal research.

#### 5.3.2 CIA Model and Drug Administration

The modeling protocol for DBA/1 mice was conducted as previously described [31]. Mice with confirmed joint swelling (day 30) were randomized into three groups: CAP‐treated (subcutaneous injection of 5% ethanol/5% Tween 80/90% saline), solvent control, and CIA model (both receiving equivalent solvent volumes). Treatments continued until day 44 euthanasia, with serum, organs, and ankle joints collected.

#### 5.3.3 Target Identification and LC–MS/MS Analysis

MH7A cells were pretreated with CAP (400 µM, 3 h) followed by CAP‐P (100 µM) or DMSO (3 h). Soluble proteins underwent click chemistry (1 mM NaVC, 100 µM THPTA, 1m M CuSO_4_, 50 µM biotin‐azide), then incubated with streptavidin beads (4 h). Beads were washed sequentially with PBS/1% SDS → 0.1% SDS → 6 M urea. Captured proteins (20–55 kDa) were Coomassie‐stained, excised, and processed with 25 mM NH_4_HCO_3_/50% ACN dehydration, followed by DTT reduction and IAA alkylation. Peptides were desalted using a C18 column, labeled with TMT 10 mass tagging reagent, and analyzed by LC–MS/MS. For pull‐down‐Western blot analysis, the abovementioned steps were performed to detect proteins on the streptavidin beads.

#### 5.3.4 Analysis of Target Proteins and Gene Ontology Enrichment Studies

P‐values for the comparisons between CAP‐P/DMSO and CAP‐P/COMPATE were calculated using a one‐sample *t*‐test based on the TMT signals from the DMSO (control), CAP‐P + CAP (COMPATE), and CAP‐P (treatment) groups. Proteins of interest were chosen based on an absolute fold change exceeding 1.2 and a *p* value below 0.05. Gene Ontology enrichment analysis of the selected proteins was performed with R (version 36).

#### 5.3.5 Coculture System

The coculture system was established following previously‐described methods [58, 59]. For RAW 264.7/RFLS coculture: RAW 264.7 cells were LPS‐primed (100 ng/mL, 3 h) before transwell coculture with RFLS ± CAP (72 h). Groups: unstimulated (blank), LPS‐only (model), LPS + CAP (treated). RFLS from upper chambers underwent qRT‐PCR. For THP‐1/MH7A system: THP‐1 cells were M1‐polarized using PMA (24 h) → IFN‐γ (20 ng/mL)/LPS (100 ng/mL) (48 h), then cocultured with MH7A ± CAP (48 h) for PCR analysis.

#### 5.3.6 Real‐Time Quantitative PCR Assay

Total RNA was extracted from the cells using Trizol, and its concentration and purity were assessed to ensure quality. The β‐actin gene served as the internal reference. Relative gene expression quantification was conducted using the 2‐ΔΔCT method.

#### 5.3.7 Immunofluorescence Staining

Fluorescence imaging was conducted as previously outlined [60]. Cells in four‐chamber dishes were treated with CAP‐P (3 h), fixed (4% PFA, 12 min, RT), permeabilized (0.2% Triton X‐100), and incubated with click chemistry reagents (2 h, RT). PRDX2 antibody (Proteintech; 1:100) was detected using fluorescent secondary antibody (1:500) and Hoechst. Images were acquired via fluorescence microscopy.

#### 5.3.8 In Situ Fluorescent Labeling Experiments

MH7A cells in 3 cm dishes were treated with CAP‐P (3 h), lysed via RIPA buffer with sonication, and quantified (BCA). Proteins underwent click reaction (RT, 800 rpm, 3 h), acetone‐precipitate, air‐dried, and analyzed by SDS‐PAGE with fluorescence scanning (Azure Sapphire). For recombinant human PRDX2 labeling: drug–protein incubation (1 h) → probe labeling (2 h) → click reaction (2 h) → 5× loading buffer heating → SDS‐gel analysis.

#### 5.3.9 Expression and Purification of Recombinant PRDX2 Proteins

WT/mutant recombinant human PRDX2 in pET28a vectors were expressed in *E. coli* BL21. Cultures grown in LB (37°C, 200 rpm) were IPTG‐induced, harvested, and lysed by sonication. Postcentrifugation (12,000×*g*, 30 min, 4°C), supernatants were buffer‐exchanged (200 mM NaCl, 20 mM Tris–HCl, 1 mM PMSF, pH8.0) for affinity purification (4 h, 4°C). Proteins were eluted via imidazole gradient, quantified (BCA), and aliquoted for downstream use.

#### 5.3.10 Assessment of Activity for Recombinant Human PRDX2 Proteins

rhPRDX2 (20 µM) was preincubated with CAP (graded concentrations, 30 min), followed by H_2_O_2_ addition (10 µL to 50 µM final). Residual H_2_O_2_ was quantified using a hydrogen peroxide assay kit (Beyotime).

#### 5.3.11 Proteomic Analysis

MH7A cells were seeded in six‐well plates and treated 24 h later for protein extraction using an SDS‐free lysis buffer. The extracted proteins were then digested into peptides and analyzed by MS. The raw data were searched using pFind and then bioinformatics analysis.

#### 5.3.12 Statistical Analysis

Statistical analysis was performed with GraphPad Prism version 9 (GraphPad, USA). Data are presented in the graphs as mean ± standard deviation (SD). Differences between experimental groups were assessed using statistical methods including one‐way ANOVA, two‐way ANOVA, Student's *t*‐test, and the rank‐sum test. A *p* value of less than 0.05 was considered statistically significant.

## Author Contributions

JG. W., Q. Z., P. L., and QF. D. designed and supervised the project. HK. H. was responsible for main experiments, data analysis, and figure production. P. L., MJ. H. and RY. H. assisted the animal experiment and performed the cellular immunostaining. MJ. H. and Y. C. performed the recombinant protein purification activity assay and molecular docking analysis. P. L. performed probe synthesis. JN. H. carried out LC–MS/MS experiments. JH. C. conducted MS data analysis. Q. Z. and QF. D. revised the manuscript. All authors read and approved the manuscript.

## Ethics Statement

All animal experiments were conducted in accordance with approved guidelines. Author: Animal Experiment Ethics Committee of Shenzhen People's Hospital. Ethics approval number: AUP‐240929‐WJG‐578‐01

## Conflicts of Interest

The authors declare no conflicts of interest.

## Supporting information



Supporting Information

## Data Availability

The mass spectrometry data generated in this study have been deposited to the ProteomeXchange Consortium via the PRIDE partner repository under accession numbers IPX0010155000 (subproject IPX0010155001, PXD057819) for TMT‐labeled quantitative proteomics data and IPX0010157000 (subproject IPX0010157001, PXD057639) for DIA‐based label‐free quantitative proteomics data. Both datasets were analyzed against the UniProt Human Proteome database.
