# Supplementary material for: Inhibition Peroxiredoxin‐2 by Capsaicin Ameliorates Rheumatoid Arthritis via ROS‐Mediated Apoptosis in Fibroblast‐Like Synoviocytes
Source: MedComm (2020). 2025 May 29;6(6):e70209. doi: 10.1002/mco2.70209 (PMC12122188; doi:10.1002/mco2.70209)
Supplement: Supplementary file 1 — Supporting Information [file MCO2-6-e70209-s001.docx]

**Supplementary Figures and Figure Legends**


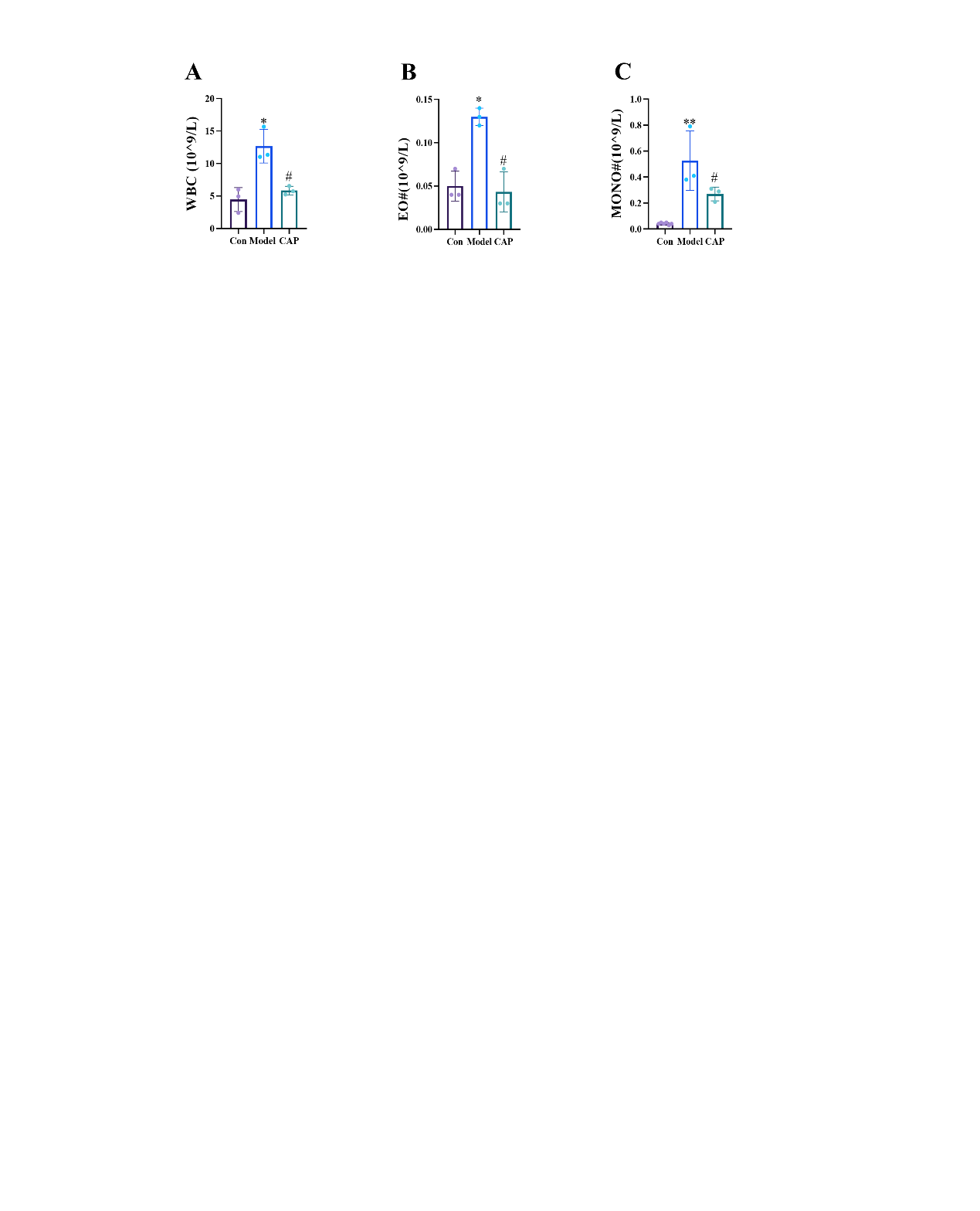


**Figure S1 Routine blood analysis of CIA mice.** White blood cells (A), eosinophils (B) and monocytes (C) from CIA mice.


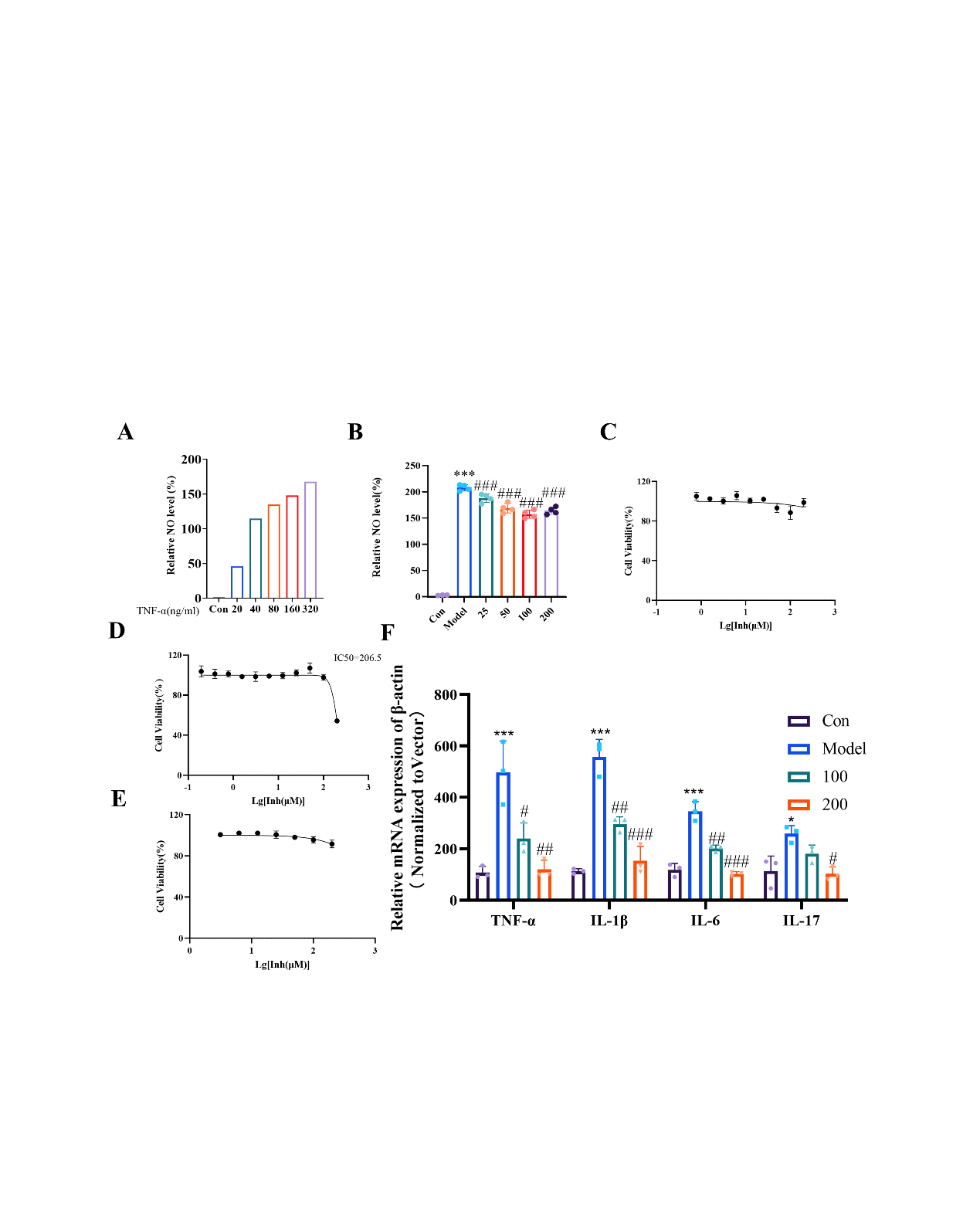


**Figure S2 (A) Relationship between TNF-α Dose and NO Release in MH7A Cells.** (B) CAP Reduces NO Levels in a Dose-Dependent Manner. (C) Effects of Different Concentrations of CAP on RAW 264.7 Cell Viability. (D) Effects of Different Concentrations of CAP on THP-1 Cell Viability. (E) Effects of Different Concentrations of CAP on RFLS Cell Viability. (F)The total mRNA level of TNF-α, IL-1β, IL-6 and IL-17 in co-culture of RAW 264.7 and RFLS cells.


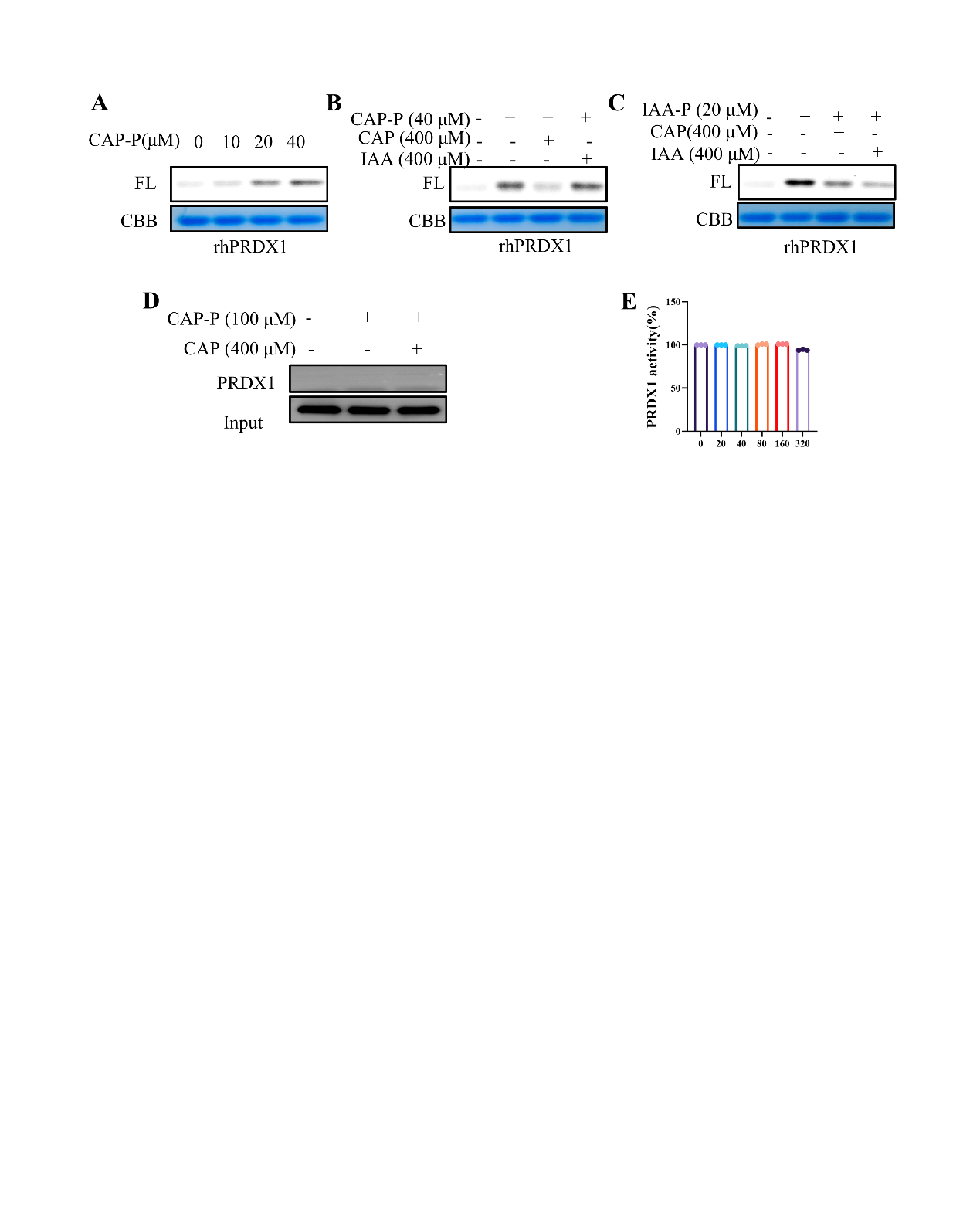


**Figure S3 Validation of CAP binding to PRDX1.** (A) The CAP-P labeling of rhPRDX1. The competition of labeling of rhPRDX1 with (B) CAP-P or (C) IAA-yne with or without unmodified capsaicin or the cysteine-alkylating agent iodoacetamide (IAA). (D)CAP-P pull-down assay, followed by western blot to confirm that capsaicin targets to PRDX1 proteins *in situ*. (F) CAP did not affect the activity of rhPRDX1 peroxide-degrading enzyme.


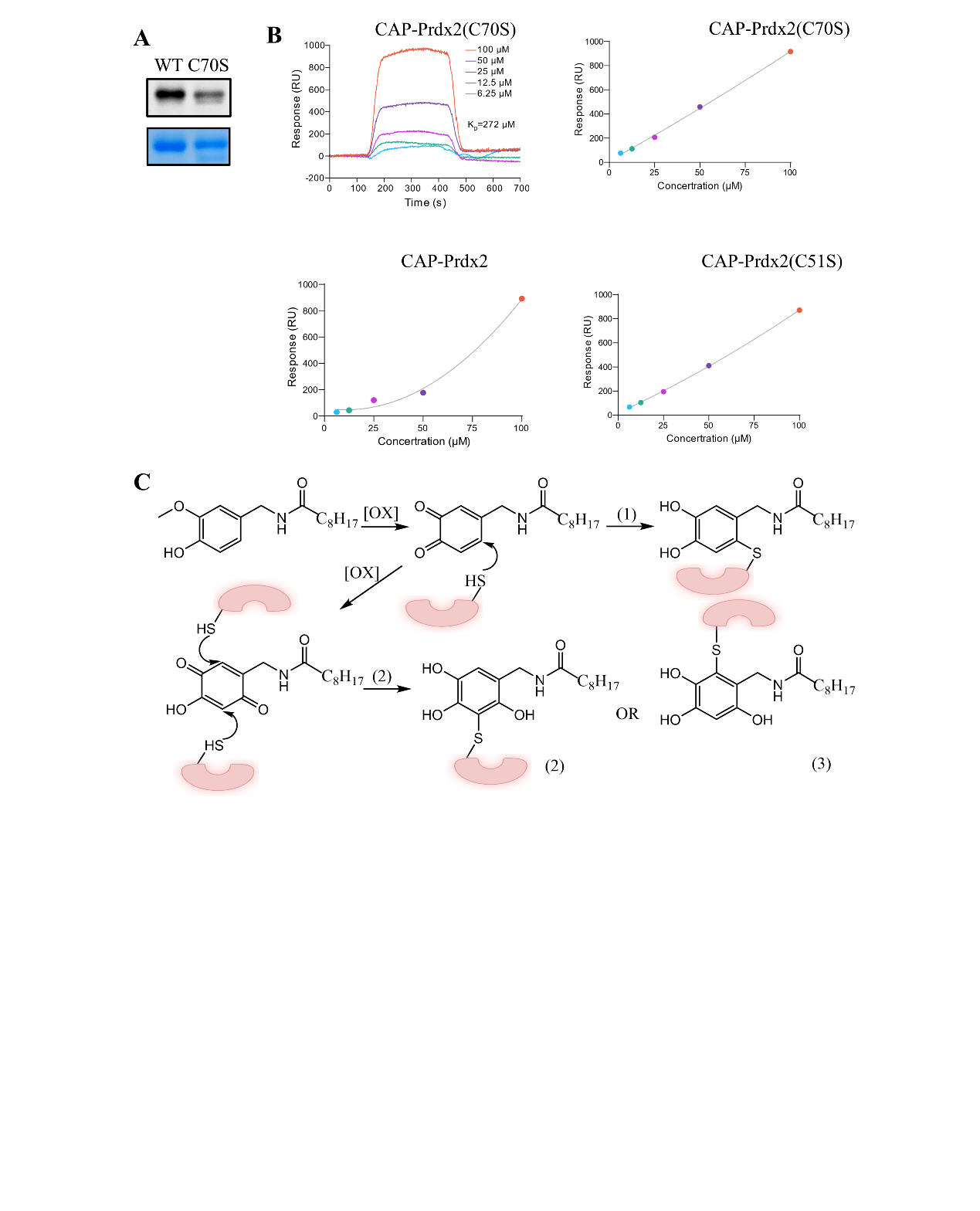


**Figure S4 Prediction of CAP binding patterns and binding sites.** (A)CAP and PRDX2-C70S labeling. (B) Supplementary Figures for SPR Experiments of CAP with PRDX2 and Various Mutants. (C) Prediction of CAP and PRDX2 binding patterns.


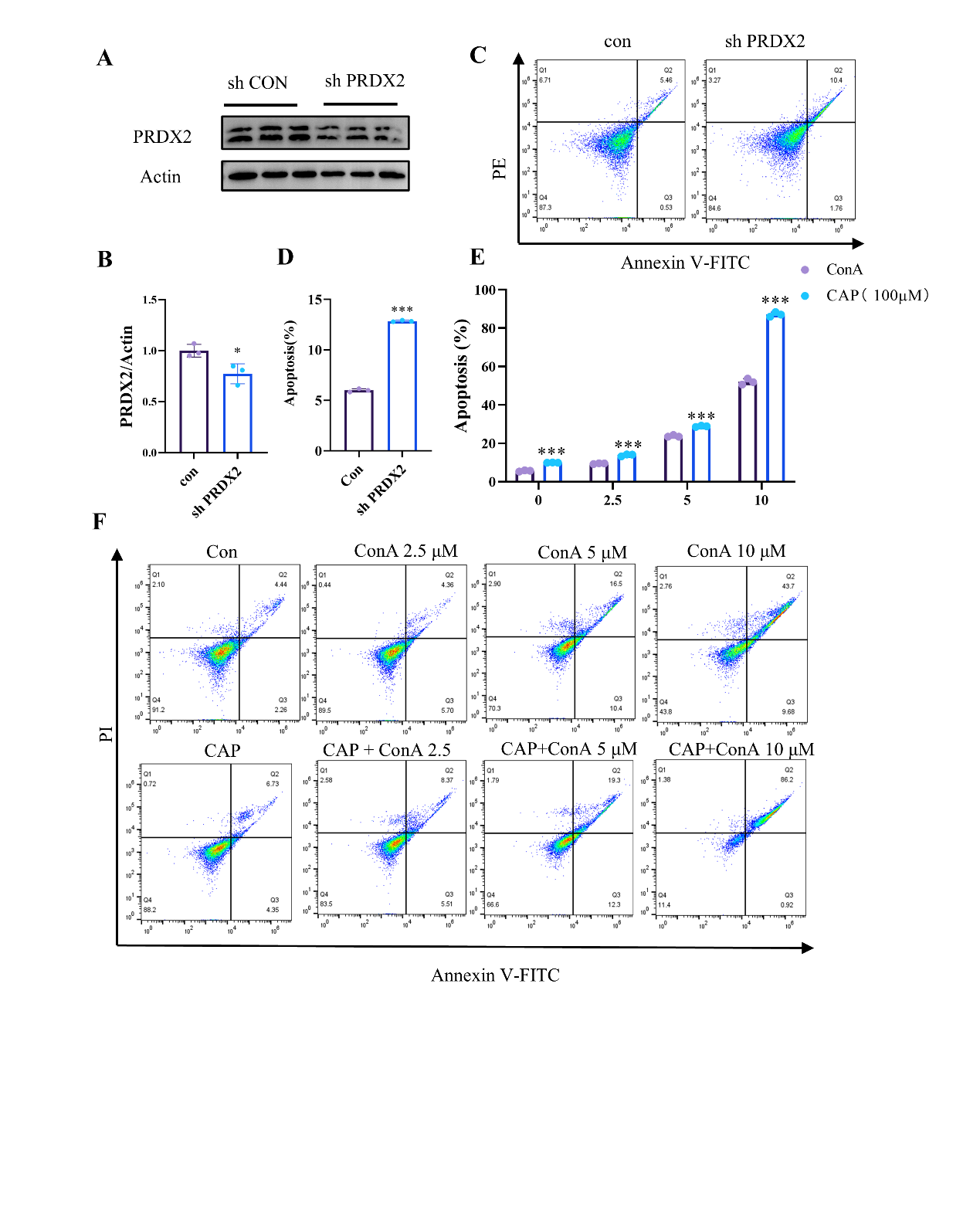


**Figure S5 CAP promoted apoptosis with binding PRDX2.** (A, B) Western blot bands and quantification for PRDX2 knockdown using shRNA. (C, D) Apoptosis flow cytometry analysis and quantification following PRDX2 knockdown. (F) Different concentrations of ConA were combined with CAP to induce apoptosis of MH7A.


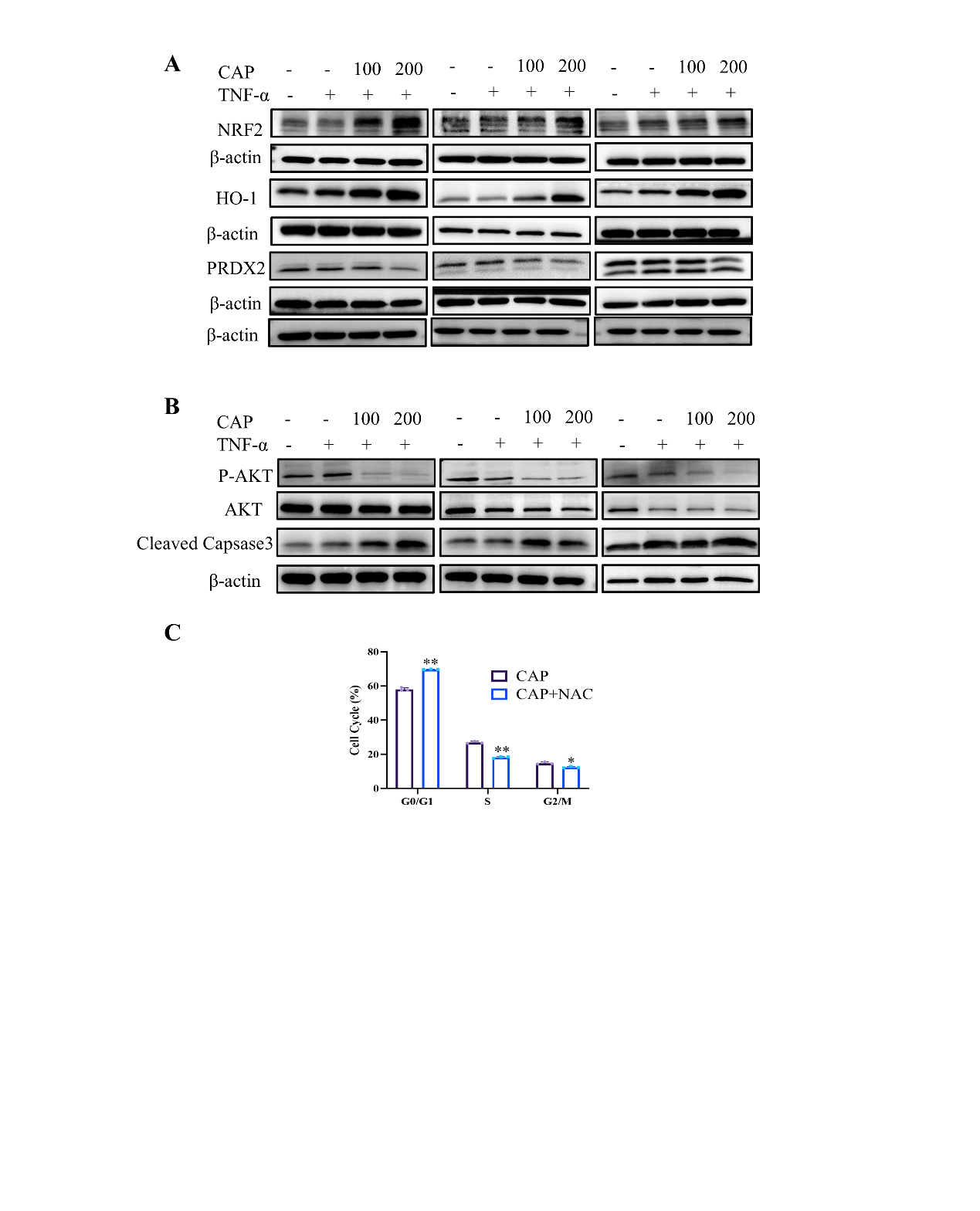


**Figure S6** **Triplicate bands for Western Blot analysis.** (A) Triplicate bands of PRDX2 and NRF2/HO-1 pathway. (B) Triplicate repeating bands of the p-AKT and Cleaved Caspase 3 apoptotic pathways. (C) Flow cytometry cycle statistics for the CAP combined with NAC group.

**Supplementary Materials and Methods**

**Detection of Cell Viability**

MH7A and RFLS cells were seeded in 96-well plates (6×10³ and 5×10³ cells/well, respectively) and treated with capsaicin or its probe at varying concentrations for 24 h. THP-1/RAW264.7 cells followed identical protocols. Cell viability was quantified via CCK-8 assay.

**Isolation of Rat Fibroblast-Like Synoviocytes**

SD rats (6-8 weeks old, male) from Vital River Laboratory Animal Technology (China) were anesthetized and euthanized. Bilateral knee joints were harvested, washed, and the synovial tissues were minced and digested with type II collagenase at 37°C for 5 hours. The resulting cells were cultured in appropriate conditions for further experiments.

**CETSA-WB**

CETSA-WB was performed per established protocols. MH7A lysates treated with CAP/DMSO (1h, RT) were aliquoted into PCR tubes, subjected to thermal gradient (37-73°C) in a thermocycler, cooled to 4°C, and centrifuged. Supernatants underwent WB to assess protein thermal stability.

**Western blotting**

The samples were incubated with the specific primary antibodies overnight and with secondary antibodies for 2 hours. An enzyme-linked chemiluminescence agent within an enhanced chemiluminescence detection system (Clinx, China) was used to detect the protein bands. Protein content was semi-quantitatively analyzed using Image J software.

**Cell scratch callus test**

MH7A cells (3×10⁵/well) were seeded in 6-well plates. Following scratch wound generation, wells were PBS-washed (×3). Cells were treated with TNF-α ± test compounds. Scratch closure was monitored by microscopy at 24 h. Wound healing was quantified using ImageJ.

**Cell migration assays**

Transwell migration assays used 24-well, 8-μm pore inserts. MH7A cells (10,000/well) in serum-free medium were seeded in upper chambers, with lower chambers containing 20% FBS as chemoattractant. Cells were treated with TNF-α ± capsaicin (CAP) for 24 h. Non-migrated cells were removed; migrated cells were fixed with 4% PFA, stained with crystal violet, and imaged. Three random fields per well were quantified using ImageJ.

**Cell invasion assays**

The bottom of the transwell chamber was coated with a layer of Matrigel. This coating simulates the extracellular matrix barrier, providing a more stringent assessment of the cells' invasive capabilities. Other conditions are the same as for migration experiments.

**Cell apoptosis assay**

MH7A cells were plated in 6-well plates with a seeding density of 1x10^6 cells per well. Twenty-four hours following drug treatment, the cells were treated according to the procedure outlined in the Annexin V-FITC/PI Apoptosis Kit manual from BD Biosciences (USA). Apoptosis was then assessed using a DXFLEX flow cytometer (USA).

**Cell cycle assay**

After 24 hours of drug treatment, the cells were processed according to the instructions provided with the Cell Cycle and Apoptosis Analysis Kit (Beyotime, China). Cell cycle analysis was then performed using a DXFLEX flow cytometer (USA).

**Molecular docking model**

The SDF file for the compound was retrieved from PUBCHEM and imported into Chemdraw 3D. The protein structure was obtained from the UniProt database and visualized using PyMOL. Subsequent processing with Mgtools 1.5.6 included dehydration, hydrogenation, charge calculation, and removal of non-polar hydrogen atoms. The ligand was then docked to the receptor using AutoDock Vina 1.1.2, and the results were visualized using both PYMOL and Discovery Studio.
